# 5′,8-Cyclopurine Lesions in DNA Damage: Chemical, Analytical, Biological, and Diagnostic Significance

**DOI:** 10.3390/cells8060513

**Published:** 2019-05-28

**Authors:** Chryssostomos Chatgilialoglu, Carla Ferreri, Nicholas E. Geacintov, Marios G. Krokidis, Yuan Liu, Annalisa Masi, Vladimir Shafirovich, Michael A. Terzidis, Pawlos S. Tsegay

**Affiliations:** 1 Istituto per la Sintesi Organica e la Fotoreattività, Consiglio Nazionale delle Ricerche, Via P. Gobetti 101, 40129 Bologna, Italy; carla.ferreri@isof.cnr.it (C.F.); annalisa.masi@isof.cnr.it (A.M.); 2 Center for Advanced Technologies, Adam Mickiewicz University, 61-614 Poznań, Poland; 3 Department of Chemistry, New York University, 31 Washington Place, New York, NY 10003-5180, USA; nicholas.geacintov@nyu.edu (N.E.G.); vs5@nyu.edu (V.S.); 4 Institute of Nanoscience and Nanotechnology, N.C.S.R. “Demokritos”, 15310 Agia Paraskevi Attikis, Greece; m.krokidis@inn.demokritos.gr; 5 Department of Chemistry and Biochemistry, Florida International University, 11200 SW 8th Street, Miami, FL 33199, USA; yualiu@fiu.edu; 6 Biochemistry Ph.D. Program, Florida International University, Miami, FL 33199, USA; ptseg001@fiu.edu; 7 Biomolecular Sciences Institute, Florida International University, Miami, FL 33199, USA; 8 Laboratory of Chemical and Environmental Technology, Department of Chemistry, Aristotle University of Thessaloniki, 54124 Thessaloniki, Greece; mterzidi@gmail.com

**Keywords:** reactive oxygen species, free radicals, DNA damage, cyclopurines, DNA and RNA polymerases, nucleotide excision repair, LC-MS/MS, xeroderma pigmentosum, cancer

## Abstract

Purine 5′,8-cyclo-2′-deoxynucleosides (cPu) are tandem-type lesions observed among the DNA purine modifications and identified in mammalian cellular DNA in vivo. These lesions can be present in two diasteroisomeric forms, 5′*R* and 5′*S*, for each 2′-deoxyadenosine and 2′-deoxyguanosine moiety. They are generated exclusively by hydroxyl radical attack to 2′-deoxyribose units generating C5′ radicals, followed by cyclization with the C8 position of the purine base. This review describes the main recent achievements in the preparation of the cPu molecular library for analytical and DNA synthesis applications for the studies of the enzymatic recognition and repair mechanisms, their impact on transcription and genetic instability, quantitative determination of the levels of lesions in various types of cells and animal model systems, and relationships between the levels of lesions and human health, disease, and aging, as well as the defining of the detection limits and quantification protocols.

## 1. Introduction

### 1.1. Reactive Oxygen Species (ROS)

Since the start of life on Earth, the human body has lived in an oxidative atmosphere due to the presence of molecular oxygen (dioxygen in its ground triplet state), which plays an immense role in biological processes [1,2]. Each individual consumes ca. 3.5 kg of molecular oxygen every day and 2.8% of it is utilized to generate free radicals. Superoxide dismutase (SOD) and nitric oxide synthase (NOS) are two classes of enzymes that control the production of superoxide radical anions (O_2_^•–^) and induce the formation of nitric oxide (^•^NO) [2]. These two radicals are the progenitors of endogenous reactive oxygen species (ROS) and reactive nitrogen species (RNS). The ROS/RNS network includes molecules such as hydrogen peroxide (H_2_O_2_), hypochlorous acid (HOCl), and peroxynitrite (ONOO^–^), as well as radicals such as hydroxyl radicals (HO^•^), nitrogen dioxide (^•^NO_2_), and carbonate radical anions (CO_3_^•–^). However, the ROS/RNS network also functions as an efficient cellular defense mechanism, being involved in the elimination of viral and microbial infections. The overproduction of ROS/RNS has been linked with the etiology of many diseases. The main processes that generate HO^•^ radicals are depicted in reactions (1)–(3): the Fenton reaction of H_2_O_2_, the reduction of HOCl by the superoxide radical anion, and the spontaneous decomposition of protonated ONOO^–^, respectively [2,3].

H_2_O_2_ + Fe^2+^ → Fe^3+^ + HO^−^ + HO^•^ (1)

HOCl + O_2_^•−^ → O_2_ + Cl^−^ + HO^•^ (2)

ONOO^−^ + H^+^ ⇆ ONOOH → ^•^NO_2_ + HO^•^ (3)

### 1.2. Reactivity of Hydroxyl Radicals with DNA

Hydroxyl radicals (HO^•^) are known for their reactivity and ability to cause DNA strand breaks and chemical modifications of nucleobase. In addition to the induction of DNA damage through the metabolism of oxygen, DNA damage may be induced through other environmentally originated insults such as ionizing radiation, UV light, and chemical mutagens. Although the majority of DNA damage induced through oxidative metabolism are single lesions, there are also types of multiple lesions, such as tandem or clustered lesions and DNA/DNA or DNA/protein crosslinking, that may challenge the repair machinery of the cell. Indeed, enzymatic systems such as base excision repair (BER) and nucleotide excision repair (NER) are known to remove the majority of DNA lesions and safeguard the integrity of the genome [4]. However, the lesions may accumulate in tissues, mainly due to the progressive loss of protective systems and consequent poor repair, as it occurs in the aging process [5]. Also, enzymatic deficiencies can give rise to the accumulation of damage to cellular components that are linked to certain pathologies.

As far as the chemical mechanisms are concerned, the site of DNA attack by diffusible HO^•^ radicals are known to be either the hydrogen atom abstraction from the 2-deoxyribose units or the addition to the base moieties, the latter being the predominant one (accounting for 85–90% of sites attacked) [6]. Moreover, experimental evidence on direct strand scission suggests the transfer of the radical center from the base moieties to the sugar backbone [6]. The order of reactivity of HO^•^ radicals towards the various hydrogen atoms of the 2-deoxyribose moiety is generally accepted to follow that of the hydrogen atom exposure to solvent (i.e., H5′ > H4’ > H3′ ≅ H2′ ≅ H1’) [7,8]. The proportion of attacks at H5′ of DNA by HO^•^ radicals is estimated to be 55% for all possible sugar positions [9]. After abstraction of a hydrogen atom from 2-deoxyribose, the fate of the carbon-centered radical depends upon the environment. There are several studies focused on the selective generation of these species to obtain quantitative data [10,11,12]. The C5′ radical has a very peculiar behavior with respect to the other positions of 2-deoxyribose, since its corresponding peroxyl radical does not generate an abasic site and leads to the formation of unique cyclic base–sugar adducts, the purine 5′,8-cyclo-2′-deoxynucleosides (cPu). These tandem-type lesions can be observed in models of DNA modifications and have also been identified in mammalian cellular DNA in vivo [13].

### 1.3. Historical Background of ‘Cyclopurines’

**Figure 1:**
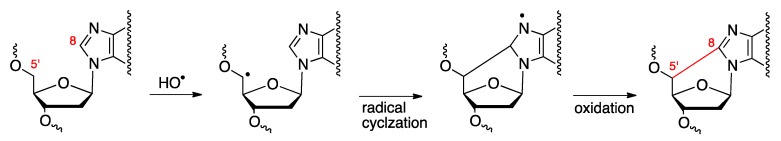
Purine 2′-deoxynucleotide reacts with a hydroxyl radical (HO^•^), yielding the purine 5′,8-cyclo-2′-deoxynucleotide via cyclization of the C5′ radical followed by oxidation.

The chemistry of purine 5′,8-cyclo-2′-deoxynucleosides (cPu) has its origin in the literature of ionizing radiation [14] and goes back to 1968, when Keck discovered that the attack of HO^•^ radicals to adenosine-5′-monophosphate leads, among other products, to the production of 5′*S* and 5′*R* diastereoisomers of 5′,8-cycloadenosine-5′-monophosphate [15]. The abstraction of the H5′ atom of the 2-deoxyribose moiety by the HO^•^ radical was suggested as the initiating step of this reaction, followed by intramolecular cyclization of the C5′ radical onto C8, leading to the formation of a new covalent bond, C5′–C8 (Figure 1).

**Figure 2:**
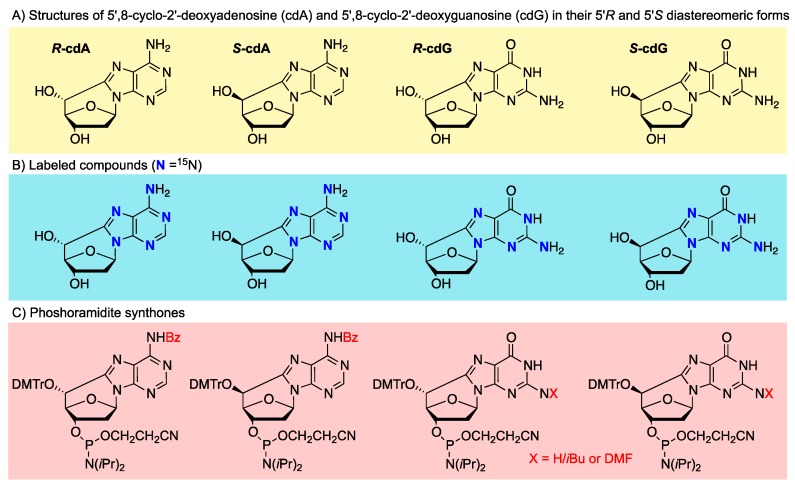
Libraries of purine 5′,8-cyclo-2′-deoxynucleosides (cPu).

In the late 1980s, Dizdaroglu and coworkers demonstrated that 5′,8-cyclo-2′-deoxyadenosine (cdA) and 5′,8-cyclo-2′-deoxyguanosine (cdG) exist in 5′*R* and 5′*S* diastereoisomeric forms (Figure 2A) and are generated by the reaction of HO^•^ radicals with the genetic material via C5′ radical chemistry of purine moieties (Figure 1) [16,17]. The induction by ionizing radiation and identification of *R*-cdG and *S*-cdG in living cells were also reported in 1987 [18].

The increasing interest in cPu lesions prompted research activity by several groups on DNA damage to investigate different areas of interest, including: nucleoside level, biomimetic models, DNA repair systems, structural investigations, biological effects, and relevance to human disease. Remarkable efforts have been made to improve the detection and quantification of the lesions in order to evaluate them in vivo and in vitro [13,19,20]. They are considered the ‘smallest’ tandem lesions and are the substrates of nucleotide excision repair (NER).

## 2. Synthesis of the cPu Library

Continuous efforts of the research community deepened our understanding of complex biological processes. When research efforts identify the structural features of specific small molecules that participate in these biological phenomena, it is of significant importance to develop synthetic procedures for generating large amounts of such molecules of interest for in-depth studies. The synthesis of molecules found in nature is also a challenge, compared to their natural synthesis, and bear important information for synthetic chemists concerning their physicochemical properties and reactivities. Bioinspired synthetic strategies are indeed being applied and have been successful in providing novel insights.

### 2.1. 5′,8-Cyclo-2′-Deoxyadenosine (cdA) and 5′,8-Cyclo-2′-Deoxyguanosine (cdG)

The existence of cdA and cdG lesions in biological systems is due to the reactions of hydroxyl radicals with the natural DNA nucleotides. A full chemical understanding of the mechanisms of formation of these lesions has been obtained by extensive exploration of the fate of the C5′ purine nucleoside radicals.

The simplest models are well understood and are based on the scenarios of reactions of 2′-deoxyadenosine (dA) and 2′-deoxyguanosine (dG) with HO^•^ radicals in the absence or presence of molecular oxygen. The two diastereomeric forms, *S*-cdG and *R*-cdG, were identified as the products of γ-irradiation of a N_2_O-saturated aqueous solution of dG; the overall reaction yields were 8–10% and the *R*/*S* product ratios were 8.3:1 (Figure 2A) [21]. In a similar experiment using dA instead of dG, two diastereomeric forms, *S*-cdA and *R*-cdA, were identified, with an *R*/*S* ratio of 6:1 and an overall yield of 10–11% [22,23]. The diastereomeric outcome is rationalized in terms of favorable hydrogen-bonded structures in the *pro*-(5′*R*) conformation. In both cases, in the presence of 2.7 × 10^–4^ M O_2_, hydrated 5′-aldehydes were formed instead of the cyclopurines, indicating that the reactions of C5′ radicals occurred with oxygen instead of cyclization [24]. The rate constants of C5′ radical cyclization at the nucleoside level were determined by pulse radiolysis studies and found to be 1.6 × 10^5^ and 6.9 × 10^5^ s^−1^ at room temperature for dA and dG, respectively [22,23,25].

**Figure 3:**
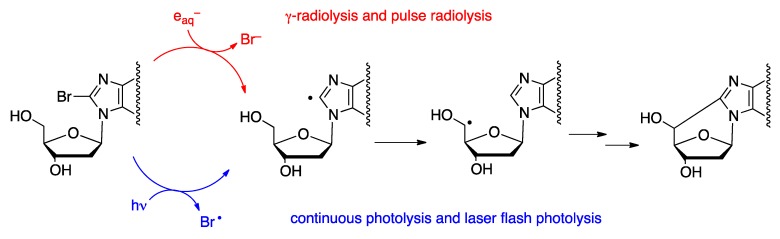
Bioinspired radical transformations for the synthesis of 5′,8-cyclopurines.

Synthetic procedures of four cPu (Figure 2A) were developed, starting from 8-bromopurine derivatives under continuous radiolysis or photolysis [21,23]. These procedures involved a radical cascade reaction that mimics the DNA damage that results in the formation of cdA and cdG lesions (Figure 3). The γ-radiolysis of aqueous solutions of 8-bromo-2′-deoxyadenosine (8-Br-2′-dA) in the presence of the K_4_Fe(CN)_6_ gives rise to the formation of cdA with an ratio *R*/*S* ratio of 6:1 and a yield of 67% (based on the starting material conversion) [22,23]. After ultraviolet light irradiation in acetonitrile, the yield of formation attained 65%, but with a diastereomeric ratio *R*/*S* of 1.7:1 [26]. The photolysis of 8-Br-2′-dG solution with ultraviolet light gave rise to cdG with a yield of 26% and an *R*/*S* ratio of 8:1 [21]. A study of the factors that influence the stability of the *pro*-5′R and *pro*-5′S conformers during the radical cyclization also revealed that the *pro*-5′R is stabilized in aqueous conditions when the 3′OH and 5′OH groups are free. The opposite behavior was observed in aprotic solvents where the *pro*-5′S conformer is favored when the 3′OH and 5′OH are coupled with relatively bulky lipophilic groups such as the TBDMS group [27,28].

The abovementioned results regarding the chemical generation and fates of the C5′ radicals in purine nucleosides led to the development of shorter procedures that afforded the four diastereoisomers of cPu in good to very good yields. Overall, starting from 8-Br-2′-dA, both *R*-cdA and *S*-cdA were obtained in good yields when the reaction was performed in aprotic solvents in the absence of oxygen using ultraviolet light irradiation. Starting with 8-Br-2′-dG, the selective protection of the free hydroxyl groups with bulky lipophilic groups such as the TBDMS group was found to improve the radical cascade of cyclization yield in aprotic solvents and shifted the *R*/*S* ratio in favor of the *S* diastereoisomer. One-step deprotection afforded both lesions in good yield. The 8-bromo derivatives are accessible following standard bromination conditions that have been reported in the literature. Thus, the C5′ radicals can be easily generated in situ by a variety of radical methodologies affording the products in good to very good yields [27,28].

### 2.2. Isotopically Labeled Derivatives

The relatively low abundance of the cPu in cellular DNA demands the development of highly sensitive analytical methods for their identification and quantification in biological samples. One of the sensitive and reliable techniques utilized by various laboratories in the field is based on liquid chromatography isotope dilution tandem mass spectrometry (see below). The synthesis of the stable ^15^*N*_5_ isotopes of the *R*-cdA, *S*-cdA, *R*-cdG, and *S*-cdG (Figure 2B) by the above-described methodologies for the synthesis of the natural lesions has been reported in the literature. In particular, photoirradiation of ^15^*N*_5_-labeled 8-Br-2′-dA in acetonitrile with UV light at *λ* = 254 nm was shown to yield the ^15^*N*_5_-labeled *R*-cdA and *S*-cdA products. The same procedure under similar conditions was also reported to give access to the ^15^*N*_5_-labeled *R*-cdG and *S*-cdG products, starting from the corresponding ^15^*N*_5_-labeled 8-Br-2′-dG [29,30]. Another synthetic strategy that involves two steps utilizes ^15^*N*_5_-labeled 2′-deoxyadenosine and ^15^*N*_5_-labeled 2′-deoxyguanosine triphosphate aqueous solutions, under γ-radiolysis conditions in the presence of nitrous oxide, to form the triphosphate 5′,8-cyclopurine intermediates. The latter are then subjected to enzymatic dephosphorylation, affording the ^15^*N*_5_-labeled cdA and cdG lesions [31]. A more straightforward methodology has been utilized for generating both *R* and *S* ^15^*N*_5_-labeled cdA lesions after γ-radiolysis of aqueous solution of ^15^*N*_5_-labeled dA in the presence of nitrous oxide [30].

### 2.3. Phosphoramidite Synthones

Further studies of the biological characteristics of the cPu lesions require the design of more complex model systems that mimic biomaterials in their natural environments. Biochemical and biological experiments that can unveil important information regarding the formation of lesions and their repair in cells are based on synthetic DNA oligonucleotides that contain lesions incorporated at specific positions in the DNA sequences according to the requirements of the experimental design. These oligonucleotide strands can be synthesized according to standard automated DNA synthesis methods.

**Figure 4:**
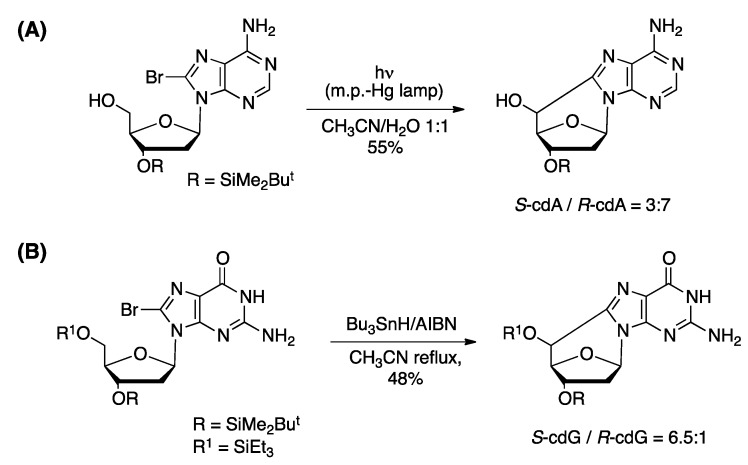
Synthesis of (**A**) 5′,8-cyclo-2′-deoxyadenosine (cdA) and (**B**) 5′,8-cyclo-2′-deoxyguanosine (cdG) in both diastereoisomeric forms, differentiating the two secondary 3′OH and 5′OH groups, by the radical cascade protocol. (m.p.: medium pressure).

The phosphoramidite synthones of the cPu that are compatible with standard automated DNA synthesis conditions can be synthesized by multistep procedures [32]. In order to achieve the synthesis of protected phosphoramidites in an appropriate manner, the 3′OH and 5′OH protecting groups should be differentiated prior to cyclization. The four phosphoramidites of *S*-cdA, *R*-cdA, *S*-cdG, and *R*-cdG (Figure 2C) were obtained in a nine-step synthesis procedure starting from the 8-Br-2′-dA or 8-Br-2′-dG, respectively, in good overall yields. In the case of the cdA series, the bromo-derivative under UV-light irradiation cyclized to give the mixture of the two diastereoisomers in 55% yield (Figure 4a). The two diastereoisomers were separated by chromatography on silica and each of them was converted to the final products following standard procedures. In the cdG series, two different protecting groups were produced for 3′OH and 5′OH moieties using deprotection with the TBDMS group, monodeprotection, and reprotection with Et_3_SiCl. This “key” derivative, cyclized under radical cascade conditions in the presence of Bu_3_SnH and AIBN as radical initiators, affords a mixture of the two diastereoisomers in good yields (Figure 4b). The two diastereoisomers were separated by chromatography on silica gel and each of these steps was followed by the necessary steps to yield the final products.

The earlier syntheses of cPu phosphoramidites based on the protocol reported by Matsuda et al. [33] for the synthesis of the *S* diastereoisomer and the subsequent inversion of the C5′ configuration were characterized by lower overall yields [34,35]. At the present time, the companies Glen Research (Sterling, VA, USA) and Berry & Associates Inc. (Dexter, MI) produce phosphoramidites of *S*-cdA and *S*-cdG. These companies commercialize *S*-cdG protected with a 5′-tetrahydropyranyl (THP) group instead of the widely used dimethoxytrityl (DMT) group. Worth noting is that the procedure used to remove the THP group is more aggressive than the one used to remove the DMT group.

## 3. Synthesis of Oligonucleotides Containing Site-Specifically Inserted cPu

The phosphoramidite derivatives were incorporated (following standard procedures, in the 3′→5′ direction [34,35]) into specific sequences of oligodeoxynucleotides (ODNs). These site-specifically modified ODNs can serve as the simplest biomimetic models of oxidized DNA lesions for studies of DNA repair pathways of cPu lesions, their biological impact, insights into structure–function relationships, and relevance of repair to the onset of human diseases. These model systems are less complex than DNA in its natural environment, but nevertheless provide valuable insights into the biological characteristics of these DNA lesions. It is worth mentioning that ODNs can also be synthesized via the automated solid DNA synthesis approach following a 5′→3′ direction by using 3′-*O*-DMTr-5′-*O*-(*β*-cyanoethyl)-*N*,*N*,-diisopropylamino-2′-deoxyribonucleoside phosphoramidites [36].

**Figure 5:**
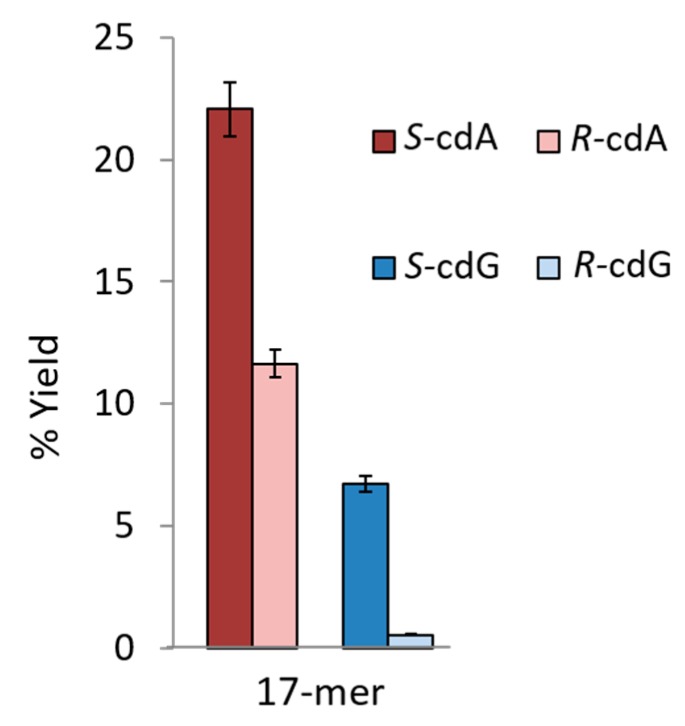
Comparison of the total yields of the 17-mer: 5′-d(CCA CCA AC**X** CTA CCA CC)-3′, where **X** = *S*-cdA, *R*-cdA, *S*-cdG, or *R*-cdG.

Figure 5 shows the results of the synthetic procedures for the same oligonucleotide sequences containing the four cPu lesions [32]. The total yields of the ODNs containing *S*-cdA or *S*-cdG were always greater than those of the ODNs containing the corresponding *R* diastereoisomers. The stereochemistry of the cPu lesions were found to influence the coupling yield and thus the total yield of the full-length modified oligonucleotides.

It is well known that the double strands have a lower molar extinction coefficient at λ = 260 nm than the corresponding mixture of single strands because the absorbance of the aromatic base system is diminished due to base–base stacking interactions in the double helix (hypochromicity). When the temperature of the sample is raised above a temperature specific for the examined length and sequence, the duplexes dissociate into single strands and the intensity of UV absorbance increases. The melting point (Tm) of an oligonucleotide duplex is defined as the midpoint of the duplex-to-single-strand transition at λ = 260 nm. In order to evaluate the structural destabilization induced by the presence of each of the four diastereomeric lesions in different oligonucleotide sequences, melting studies were carried out, in the absence and presence of cPu lesions, by several research groups (Table 1). The thermodynamic destabilization of the duplexes containing the lesion was observed by the decrease of the melting points of modified duplex sequences with respect to unmodified ones. In the case of 17-mer double-stranded (ds) oligonucleotides (Table 1), it was demonstrated that all four cPu lesions destabilize identical duplex sequences, with cdA producing more destabilization than cdG. Shorter DNA duplexes are more easily destabilized by the lesions than longer ones.

The presence of the C5′–C8 bond in cPu lesions causes substantial structural changes in DNA. These structural changes include displacement of the purine base, an unusual sugar pucker, deformation of the sugar–phosphate backbone, and alterations in the base stacking with adjacent nucleotides in DNA, especially the 5′ side relative to the lesion [37,38,39,41,42]. Molecular dynamics simulations were also used by several groups to evaluate the structural distortions and dynamics induced by cPu lesions in comparison with unmodified DNA. It was found that the *R* diastereomers cause a greater backbone distortion and base stacking impairment than the *S* diastereomers [37,38,39,43]. The differences in the structural perturbation effects, as caused by the presence of the *R* and *S* diastereoisomers, provide some rationale for the observation that the *R* isomers are more efficiently recognized by the nucleotide excision repair (NER) system [37,39,44].

## 4. Base Excision Repair (BER) and Nucleotide Excision Repair (NER) Pathways

The stability of the human genome subjected to oxidative stress is maintained by the repair of oxidatively generated DNA lesions in cellular DNA [45]. Non-bulky oxidatively generated DNA lesions are typically repaired by BER mechanisms [46] that are highly conserved in all three domains of life: Bacteria, Archaea, and Eukarya [47,48]. The BER proteins bind to the damaged nucleotide and induce cleavage of the *N*-glycosyl bond, thus forming abasic sites. In the case of monofunctional glycosylases, abasic sites are cleaved by an apurinic (AP) human endonuclease (APE1) that results in the formation of fragments with 3′-OH and 5′-deoxyribose phosphate (5′-dRP) at the ends [49]. In turn, bifunctional glycosylases cleave abasic sites by AP lyase activities that result in the formation of single-strand breaks containing either a phosphate (P) group (β, δ-elimination), or an α,β-unsaturated aldehyde (PUA, β-elimination) at the 3′-end [50,51]. Thus, the BER mechanism results in the excision of the damaged nucleotide and the formation of single-strand breaks that can be detected by gel electrophoresis methods [52]. Brooks et al. [36] have shown that cdA lesions are not repaired by BER pathways in adult rat brain nuclear extracts [53], although other known BER substrates [54] were efficiently removed. Furthermore, none of the cPu lesions *R*-cdA, *S*-cdA, or *S*-cdG were found to be substrates of DNA glycosylases in HeLa cell extracts [44]. Pande et al. [55] also reported that the seven purified BER proteins (*E. coli* Fpg, Endo III, Endo V, and Endo VIII; human OGG1; human NEIL1; and NEIL2) neither bind to nor excise *S*-cdA or *S*-cdG lesions from double-stranded (ds) oligonucleotide duplexes. In summary, these results clearly demonstrate that the cPu lesions are not substrates of BER pathways.

The human NER machinery typically excises bulky DNA lesions, such as those derived from the binding of metabolically activated polycyclic aromatic hydrocarbons (PAH) to DNA [56]. Bulky DNA lesions are recognized by the DNA damage sensor and NER factor XPC-RAD23B, a heterodimeric protein complex. After binding to the DNA lesion, the ten-protein NER factor TFIIH, XPA, and the endonucleases XPF-ERCC1 and XPG are recruited to the XPC–TFIIH–DNA lesion complex. The two endonucleases incise the damaged strand on the two sides of the bulky DNA lesion, thus excising the characteristic ~24–30 nucleotide (nt) dual incision products that contain the lesion and are the hallmarks of successful NER [57,58].

More recently, it was demonstrated that certain non-bulky DNA lesions, such as the oxidatively generated diastereomeric spiroiminodihydantoin (Sp) and 5-guanidinohydantoin (Gh), are the substrates of both BER and NER pathways in human cell extracts [59] and in intact human cells [60]. In contrast, cPu lesions are repaired exclusively by the NER pathway [13,19,61].

Employing the host cell reactivation assay, Brooks et al. [36] found that the *S*-cdA lesions strongly block gene expression in Chinese hamster ovary (CHO) cells and in SV40-transformed human fibroblasts. Furthermore, the repair of cdA lesions was significantly suppressed in NER-deficient CHO cells and in human cells from patients who are carriers of XP complementation group A (XPA) mutations that are associated with neurodegeneration [36]. NER dual incision products were also detected after incubation of plasmid DNA harboring *R*-cdA or *S*-cdA lesions in HeLa cell extracts [44]. The NER incision of *R*-cdA was more efficient than in the case of *S*-cdA. In cell extracts supplemented with antiserum against XPA, the NER dual incision efficiency was significantly reduced; in turn, NER activity was restored by the addition of purified XPA protein [44]. The time course of dual incisions from 136 bp DNA duplexes harboring *S*-cdA and *S*-cdG lesions in HeLa cell extracts was compared to the rate of excision of *cis-anti*-B[*a*]PDE-dG adducts (an excellent substrate of human NER [62]) embedded in an identical sequence context in HeLa cell extracts; it was shown that the *cis-anti*-B[*a*]PDE-dG adduct (where B[*a*]PDE-dG denotes the product of reaction of the B[*a*]P diol epoxide metabolite (+)-7*R*,8*S*-dihydrodiol, 9*S*,10*R*-epoxy-tetrahydrobenzo[*a*]pyrene, BPDE) with the exocyclic amino group of guanine in double-stranded DNA was repaired more efficiently than the *S*-cdG lesion. In turn, the latter was a better NER substrate than the *S*-cdA lesion embedded in the same sequence context under identical conditions [55].

The relative NER efficiencies of all four cPu in the same sequence context were measured and compared in human HeLa cell extracts for the first time under identical conditions [39]. The cdA and cdG lesions were excised with similar efficiencies, but the NER excision rates measured for both *R* diastereoisomers were greater by a factor of ~2 than in the case of the *S* lesions. Molecular modeling and molecular dynamics simulations revealed the structural and energetic origins of this difference in NER-incision efficiencies. The characteristic C5′–C8 bond in both kinds of diastereoisomeric cPu lesions causes a greater local distortion of the DNA backbone and a greater disruption of local van der Waals stacking interactions in the case of the *R* than the *S* diastereoisomeric cdA and cdG lesions. Molecular dynamic simulations indicate that the local structural dynamic fluctuations are more pronounced in the case of the diastereoisomeric *R* than the *S* cPu lesions [39]. Therefore, the greater local dynamics and destabilization of stacking interactions associated with the *R* diastereoisomers appear to be correlated with their higher susceptibilities to NER compared to the *S*-cdA and *S*-cdG lesions.

**Figure 6:**
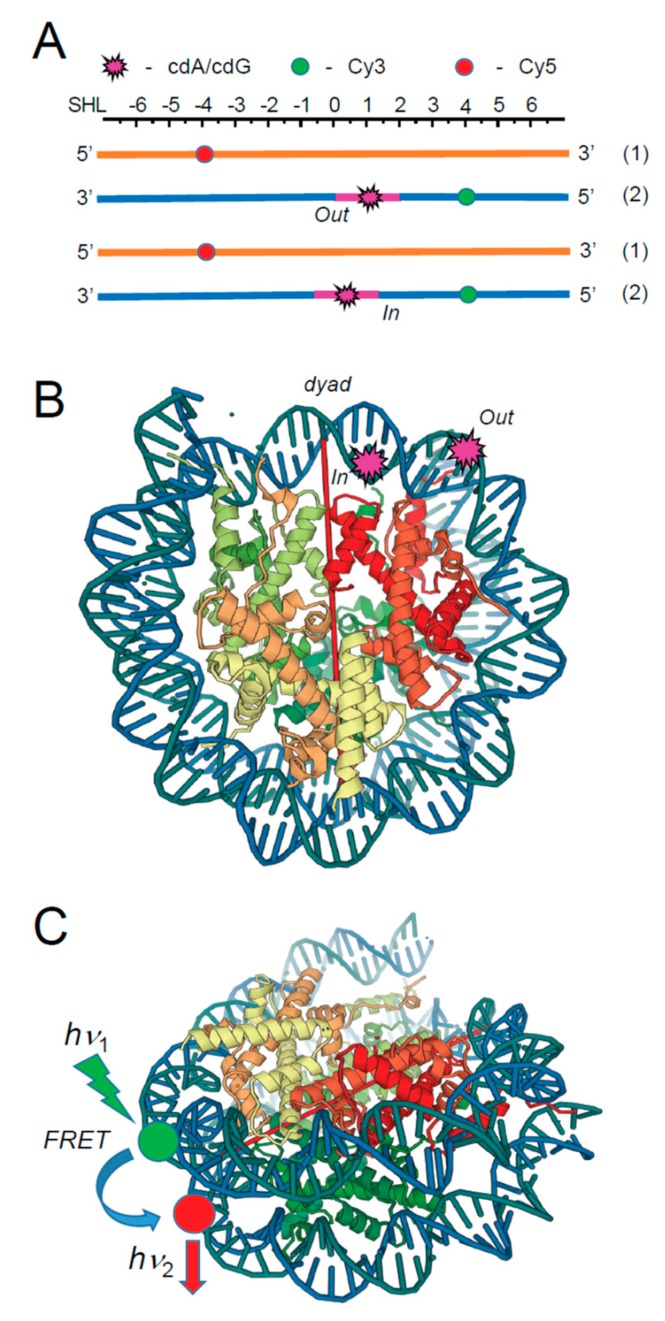
(**A**) Schematic illustrations of the placements of the DNA lesions and the Cy3 donor and Cy5 acceptor molecules at the “*Out*” and “*In*” rotational settings in the 147-mer 601 DNA duplexes used in the Förster Resonance Energy Transfer (FRET) experiments. (**B**) The crystal structure of the 601 nucleosome core particles (PDB 3LZ0) [67]. The dyad axis is indicated by the red line. (**C**) Positions of the Cy3 donor and Cy5 acceptor molecules in the nucleosome FRET experiments. The lesions were positioned at the 66th or 70th nucleotide (nt) counted from the 5′-end of the 147-mer, corresponding to the *Out* or *In* rotational settings. The internal Cy3 and Cy5 labels were positioned at nucleotides 43 and 39 counted from the dyad axis in opposite strands. Reproduced from reference [66].

**Figure 7:**
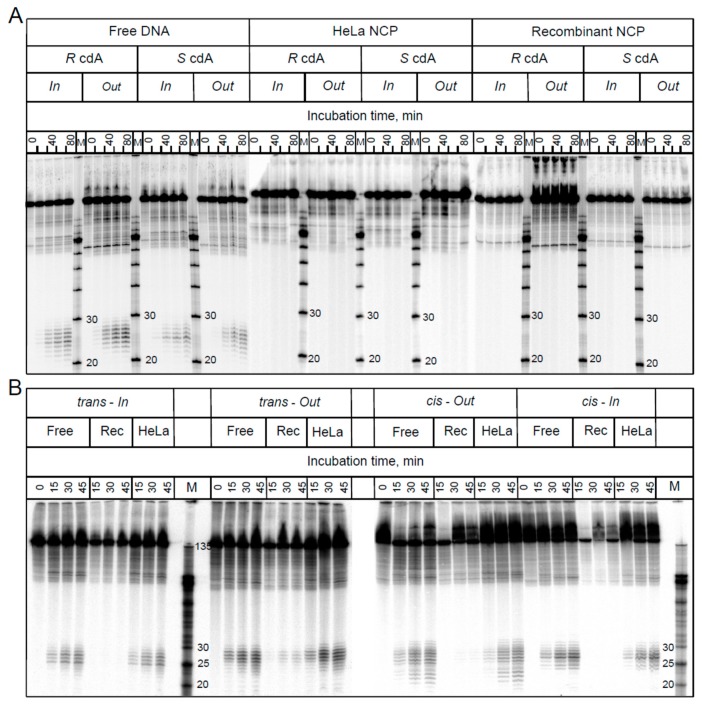
Representative autoradiographs of denaturing gels of the results of nucleotide excision experiment assays in HeLa cell extracts. (**A**) The substrates were either free 147-mer 601^cP^ DNA sequences containing single cPu lesions or nucleosomes assembled with histone octamers derived from recombinant (Rec) histones or native, post-translationally modified histones extracted from HeLa cells. (**B**) Analogous nucleotide excision repair (NER) experiments with *cis*- and *trans*-B[a]PDE-dG adducts positioned at the same *In* and *Out* superhelical locations. Three separate gels are depicted in panels A and B. Reproduced from reference [66].

The relative NER efficiencies at the level of chromatin or nucleosomes, the primary subunits of chromatin, can be significantly reduced to those of free or naked DNA because DNA lesions embedded in nucleosomes are less accessible to NER repair protein factors [63,64,65]. To explore the effects of nucleosome environments on the relative NER efficiencies, all four cPu were embedded at the *In* or *Out* rotational setting near the dyad axis in nucleosome core particles reconstituted either with native histones extracted from HeLa cells (HeLa-NCP) or with recombinant histones (Rec-NCP), as shown in Figure 6 [66]. These experiments showed that while the cPu and B[*a*]PDE-dG lesions in free DNA are good NER substrates, the non-bulky cdA and cdG lesions embedded at either the *In* or *Out* rotational setting of native HeLa histone-derived nucleosomes are completely resistant to NER in human cell extracts (Figure 7).

By contrast, the relative excision rates of the *trans*- and *cis*-B[*a*]PDE-dG adducts that are excised at different rates in free DNA are reduced by the same factor of ~2.2 in HeLa nucleosomes and by the much greater factor of ~11 in recombinant histone nucleosomes. Molecular dynamics simulations showed that the *cis-anti*-B[*a*]PDE-dG adduct is more dynamic and more destabilizing than the smaller and more constrained cdG lesions, suggesting more facile access to the bulkier *cis-anti*-B[*a*]PDE-dG lesion [68]. By contrast to the bulky B[*a*]PDE-dG adducts, the cPu lesions embedded in the same sequence contexts in either post-translationally modified HeLa histone-derived nucleosomes or unmodified recombinant histone nucleosome core particles are fully resistant to NER in human cell extracts.

The NER response of the B[*a*]PDE-dG adducts in HeLa-NCPs is not directly correlated with the observed differences in the thermodynamic destabilization of HeLa NCPs, the Förster resonance energy transfer (FRET) values, or hydroxyl radical footprint patterns and is weakly dependent on the rotational settings [66]. These and other observations suggest that NER is initiated and limited by the binding of the DNA damage-sensing NER factor XPC-RAD23B to a transiently opened B[*a*]PDE-dG-modified DNA sequence in HeLa histone nucleosome particles that corresponds to the known footprint of XPC–DNA–RAD23B complexes (≥30 base pairs). These observations are consistent with the hypothesis that post-translation modifications and the dimensions and properties of the DNA lesions are the major factors that have an impact on the dynamics and initiation of NER in nucleosomes. These results further suggested the hypothesis that the non-bulky cPu DNA lesions do not sufficiently perturb the dynamics of nucleosomes and are therefore resistant to NER in human cell extracts [66].

It remains unknown whether these observations carried out with post-translationally modified and recombinant histone-derived nucleosome particles in vitro are also relevant to the same lesions embedded in chromatin in intact human cells and tissues. It is therefore important to extend such studies to the more challenging in vivo environments in order to determine whether the physically smaller cPu lesions are also more resistant to repair in their natural chromatin settings than lesions derived from the binding of metabolites of bulky polycyclic compounds to native DNA in vivo.

## 5. Bypassing of cPu Lesions by DNA and RNA Polymerases and the Resulting Biological Consequences

**Figure 8:**
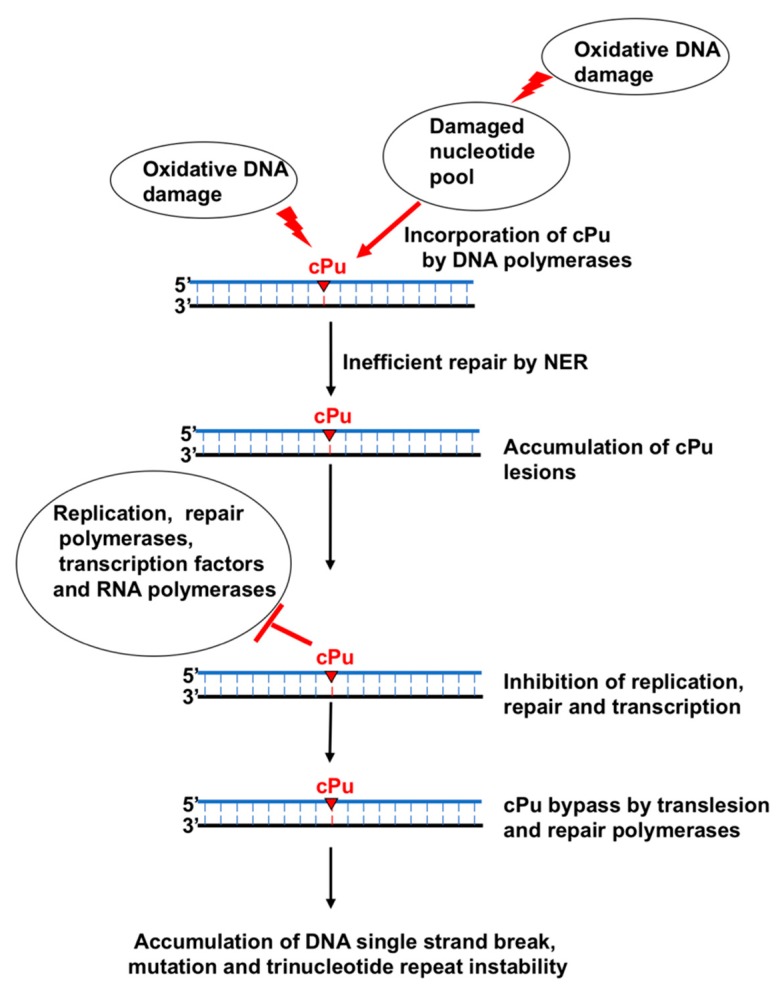
Accumulation of cPu disrupts DNA replication, repair, and gene transcription, leading to lesion bypass, mutations, and genome instability.

As already discussed, unlike other oxidized DNA base lesions, cPu lesions cannot be repaired by the BER pathway. This results in the accumulation of the lesions in the genomic DNA and distorts the DNA backbone, initiating the NER pathway. On the other hand, NER repairs cPu lesions at low efficiency compared to its repair of other bulky DNA lesions, thereby resulting in the accumulation of cPu lesions in DNA. When DNA and RNA polymerases encounter the lesions during DNA replication and repair and gene transcription, they have to bypass the lesions to complete the biological processes (Figure 8). Studies have shown that repair DNA polymerases such as DNA polymerase β (pol β) and translesion DNA polymerases, including pol η and ι, and ζ, can bypass cdA [69,70,71]. Also, cdA lesions can be bypassed by an RNA polymerase [36,72]. A study from the Kuraoka group has found that *E. coli* polymerase I (pol I) can incorporate *R*-cdA and *S*-cdA into DNA [73].

### 5.1. cPu Incorporation by a Replicative DNA Polymerase

The Kuraoka group found that the *E. coli* DNA polymerase I (pol I) large protein fragment, the Klenow fragment, which lacks 5′–3′ exonuclease activity [74], can incorporate 5′*R* and 5′*S* diastereoisomers of cdATP at different efficiencies [73]. The incorporation of *R*-cdATP and *S*-cdATP into a base pair with a dTMP by the pol I fragment is about 17,000-fold and 750-fold less efficient than that of dATP, respectively. The rate of the incorporation of *S*-cdATP and *R*-cdATP opposite to a dTMP by the Klenow fragment is 25.6 µM^−1^ min^−1^ and 1.13 µM^−1^ min^−1^, respectively. The extension of a cdA by the pol I fragment is only slightly inhibited by the two isomers of cdA, although *S*-cdATP is more readily incorporated and extended by the Klenow fragment [73]. The pol I Klenow fragment incorporates *S*-cdATP more efficiently than *R*-cdATP. This is because the active site of the Klenow fragment binds to *R*-cdA and *S*-cdA with a different affinity due to the stereospecific difference between the two isomers. By superimposing *R*-cdATP and *S*-cdATP on the incoming dATP, the interaction between the Klenow fragment and the cdA lesions is revealed. It is found that the 5′-phosphate group of the *R*-cdA is turned away from the active site of the polymerase. In contrast, the 5′-phosphate of the *S*-cdA turns toward the active site [73]. Thus, compared to *S*-cdATP, the *R*-cdATP can barely form a hydrogen bond with dTMP through the transition from an opened to a closed conformation in the active site of the polymerase, thereby leading to low efficiency of its incorporation. Interestingly, both stereoisomers of cdATP can also be incorporated by the polymerase to base-pair with dCMP. However, *R*-cdATP preferentially base-pairs with dCMP rather than dTMP, suggesting that *R*-cdATP is more error-prone than *S*-cdATP.

### 5.2. Inhibition of Gene Transcription by a cPu Lesion and Its Bypass by an RNA Polymerase

A cdA lesion can also inhibit the binding of TATA box binding protein (TBP) [75] and RNA polymerase II to the CMV promoter that regulates the luciferase reporter gene [36], resulting in reduced synthesis of RNA that further decreases the luciferase reporter gene expression [36,72,75]. It has been shown that XP cells transfected with plasmids containing a single *S*-cdA lesion located at the second A in the TATA box of the CMV promoter exhibit 75% reduced luciferase gene expression [75]. In a study that has also tested the effects of *S*-cdA on the transcriptional activity of RNA polymerase II, XP cells were transfected with a plasmid carrying a single *S*-cdA lesion in the transcribed region of the luciferase reporter gene. The results show that XP cells transfected with plasmids containing the lesion still exhibit 20–30% of the luciferase activity of the XP cells transfected with the plasmids without a lesion [36]. This indicates that the presence of an *S*-cdA does not entirely abolish the activity of RNA polymerase II, further suggesting that RNA polymerase can partially bypass an *S*-cdA in the template, and the bypass of a cdA by RNA pol II can result in full-length transcribed products. It has been found that yeast RNA pol II bypasses a cdA by preferentially incorporating UTP that base-pairs with a cdA, although it also misincorporates rA, rG, and rC to base-pair with the lesion with low efficiency [72]. In the presence of ATP alone, yeast RNA pol II can efficiently incorporate it to base-pair with a cdA, but with a much lower rate than its incorporation of UTP. To continue to extend the nucleotide that base-pairs with a cdA, RNA pol II can incorporate an rA opposite a dA next to the lesion. Besides, the transcription initiation/elongation factor TFIIF can stimulate the activity of RNA pol II of bypassing a cdA lesion without affecting its fidelity, indicating that the *cis* and *trans* factors can also affect the efficiency of the bypassing of a cPu lesion by RNA pol II [72].

### 5.3. Inhibition of DNA Polymerase Activities by a cPu Lesion and Its Bypass by DNA Polymerases

A cPu lesion can alter the activity of DNA polymerases. It has been reported that the DNA synthesis activities of calf thymus replicative DNA polymerases, such as pol δ, and the bacterial phage DNA polymerase T7 DNA polymerase are inhibited completely by *R*-cdA and *S*-cdA lesions, resulting in replication fork stalling [44]. The primer extension activity of calf thymus pol δ is abolished at an *R*-cdA or *S*-cdA lesion site in the template strand, whereas T7 DNA polymerase can manage to extend the primer at the cPu lesions [44]. This indicates that the DNA synthesis of the pol δ ceases before the cPu lesions, while T7 DNA polymerase can bypass *R*-cdA or *S*-cdA by incorporating an additional nucleotide. T7 DNA polymerase bypasses an *R*-cdA more efficiently than an *S*-cdA. The results indicate that cPu lesions can lead to DNA replication stalling by inhibiting the activities of replication DNA polymerases, further suggesting that the lesions have to be bypassed by translesion DNA synthesis in cells to resolve the stalled replication fork and restart DNA replication.

An *S*-cdA lesion located in double-stranded DNA can also inhibit pol β DNA synthesis and BER in cell extracts during the repair of an abasic (AP) site at the complementary strand [76]. The inhibitory effect on the DNA synthesis activity is determined by the location of the AP site relative to the *S*-cdA lesion. The DNA synthesis at the AP site located at the 5th or 8th nucleotide upstream (−8 or −5) or downstream (+5 or +8) of *S*-cdA is not significantly affected [76]. However, the polymerase only can exhibit minimal incorporation of a nucleotide at the AP site located at the 1 nucleotide upstream (−1) and downstream (+1) of and opposite to (0) the *S*-cdA [76]. These indicate that an *S*-cdA lesion close to an abasic site can inhibit the DNA synthesis by the DNA polymerase during BER, whereas the lesion located at ≥5 nt away from an AP site does not affect its DNA synthesis. It is suggested that an *S*-cdA induces a geometric alteration of the DNA surrounding the AP site, and this may inhibit the binding of pol β to the DNA, thereby inhibiting its DNA synthesis.

The Basu group has further identified a translesion DNA polymerase that can bypass a cPu lesion in *E. coli*. They demonstrate that *S*-cdA and *S*-cdG strongly block *E. coli* replicative and repair DNA polymerases, including pol II, Klenow fragment, pol IV, and pol V, as well as *S. solfataricus* P2 DNA polymerase IV (Dpo4) [77,78]. Through the gene knockout of pol II, pol IV, and pol V in the SOS-induced or uninduced *E. coli* strains, the group has found that pol V is the one that is responsible for bypassing of *S*-cdA or *S*-cdG inserted in a plasmid through translesion DNA synthesis in *E. coli*, whereas pol II and pol IV do not play a role in bypassing of the lesions. This is further supported by the results showing that the *E. coli* strain with pol V deficiency that bears the plasmids containing an *S*-cdA or *S*-cdG cannot survive [77,78]. This demonstrates that pol V is required for the bypass of *S*-cdA or *S*-cdG in *E. coli*. However, in vitro biochemical characterization has shown that the *E. coli* Klenow fragment, pol IV, and Dpo4 can incorporate nucleotides to base-pair with *S*-cdA or *S*-cdG [78]. Specifically, the Klenow fragment preferentially incorporates dTTP and dCTG to base-pair with *S*-cdA or *S*-cdG, respectively. On the other hand, biochemical characterization indicates that pol IV can incorporate dTTP and dCTP to base-pair with *S*-cdA and dCTP to base-pair with *S*-cdG. Dpo4 can insert dTTP and dGTP to base-pair with *S*-cdA. However, it preferentially inserts dTTP over dCTP to base-pair with *S*-cdG. This may be due to a more opened and less rigid active site in Dpo4 polymerase that can tolerate distorted and bulky DNA lesions. A study conducted by Xu et al. has also shown that the bypass of *S*-cdG by *S. solfataricus* Dpo1 and Dpo4 is decreased by 140- and 65-fold compared to their bypass of dG [79]. The authors further demonstrate that Dpo1 and Dpo4 preferentially incorporate dCTP opposite to *S*-cdG. In addition, the polymerases can misincorporate dATP and dTTP opposite to the lesion [79]. The results of Dpo4 differ from those reported by Basu’s group, which showed that Dpo4 preferentially incorporates dTTP opposite to *S*-cdG [78]. The inefficient bypass of *S*-cdG by Dpo1 and Dpo4 appears to result from their poor DNA synthesis at the lesion, with the nucleotide incorporation reduced by 200- and 3000-fold [79]. On the other hand, the binding affinity of Dpo1 and Dpo4 to a substrate containing *S*-cdG is either not affected or moderately reduced by only 6-fold [79]. The structural base of the DNA polymerases has been revealed by the results from a crystal structure containing a Dpo4:*S*-cdG:dCTP complex and showing that *S*-cdG is shifted to the major groove of the DNA substrate. This separates the α-phosphate dCTP from the C3′ atom of the primer terminal dideoxy C (C^dd^) by 9.2 Å, thereby preventing nucleotidyl transfer activity of the polymerase for catalyzing the DNA synthesis [79]. In a crystal structure of the Dpo4:*S*-cdG:dTTP complex, the formation of a looped template has been also identified. This suggests that Dpo1 and Dpo4 can skip the lesion by looping out the template strand. This may further lead to sequence deletion, thus providing the structural evidence for the findings about a cPu-induced repeat sequence instability reported by the Liu laboratory [69]. These results indicate that the Klenow fragment, pol IV, Dpo1, and Dpo4 can manage to bypass cPu lesions. The results further indicate that pol IV, Dpo1, and Dpo4 are more error-prone than the Klenow fragment by performing nucleotide misincorporation to bypass a cPu lesion.

In eukaryotic cells, cPu lesions can also be readily bypassed by several human and yeast translesion DNA polymerases, pol η, pol ι, and pol ζ, but not pol κ. These DNA polymerases can incorporate different nucleotides to base-pair with an *S*-cdA and *S*-cdG [70]. For example, pol ι can incorporate dTTP, dGTP, and dATP to base-pair with *S*-cdA, whereas it incorporates dCTG, dATP, and dGTP to base-pair with *S*-cdG. In contrast, human pol η usually incorporates a correct nucleotide to base-pair with *S*-cdA or *S*-cdG, whereas yeast pol η can also misincorporate dTTP opposite to *S*-cdG [80]. Among these DNA polymerases, pol η and pol ζ can extend the nucleotides incorporated opposite an *S*-cdA or *S*-cdG lesion [70,80]. Human pol η can only extend a matched nucleotide that base-pairs with a cPu lesion. However, yeast pol η can extend both matched and mismatched nucleotides that base-pair with the lesions [80]. It is proposed that human pol η, pol ι, and pol ζ cooperate to bypass cPu lesions during DNA replication and repair. Cell-based mutation analysis has further demonstrated that the bypass of cPu lesions through these translesion DNA polymerase results in a wide spectrum of mutations, indicating the misincorporation of nucleotides through the bypass of cPu lesions in cells [70].

A recent study from the Yang group has further revealed the molecular basis underlying the bypass of an *S*-cdA by human pol η using the cocrystals of pol η and the DNA substrates containing an *S*-cdA lesion [81]. The crystal structures indicate that the C5′–C8 covalent bond of cdA distorts the backbone of the DNA template by shifting the sugar toward the minor groove. This further results in the change of the width of duplex DNA and pushes the adenine of the cdA to be tilted toward the 5′–direction, leading to the disruption of the base stacking between the damaged nucleotide and the adjacent base. The structural study further reveals that ~60% of adenines from the damaged nucleotide are shifted into the major groove, thereby preventing the formation of a hydrogen bond between the damaged nucleotide and dTTP. In addition, the presence of different type of metal ions can also alter the configuration of the active site, either facilitating the incorporation of dTTP opposite cdA or preventing the formation of the hydrogen bonds between cdA and dTTP. The effect is also mediated through the opening of the finger domain of pol η to accommodate the DNA backbone distortion. Since cdA is shifted to the major groove, this protects it from forming the hydrogen bonds with dT at the 3′-end. Instead, this allows cdA to make only van der Waals interaction with the dT, preventing the formation of a new base pair with the incoming nucleotide and primer extension. Thus, as a result, pol η fails to extend the dT opposite the cdA lesion. The study provides novel insights into the structural basis underlying the nucleotide incorporation by pol η in bypassing a cdA lesion [81].

Similar to the Y family translesion DNA polymerases, DNA polymerase β (pol β), a central component of BER [82,83], can also bypass a cdA lesion [84]. Pol β can readily bypass both *R*-cdA and *S*-cdA located in the substrates mimicking DNA replication and BER intermediates [84]. It has been shown that pol β wild-type mouse embryonic fibroblast (MEF) cell extracts can generate a significant amount of DNA synthesis products resulting from the bypass of an *R*-cdA and *S*-cdA lesion located in an open template, i.e., a DNA substrate containing a 1-nt gap or 1-nt gap with a sugar–phosphate residue [84]. However, pol β-knockout MEF cell extracts generate only a small amount of the lesion bypass products on all the substrates [84]. The results suggest that pol β also plays an important role in bypassing a cPu lesion during DNA replication and repair in mammalian cells. Further biochemical analysis has shown that pol β mainly incorporates a dT to base-pair with a *R*-cdA, but can also misincorporate dA, dG, and dC to base-pair with the damaged nucleotide at low efficiency. Pol β only inserts a dT opposite an *S*-cdA lesion. Moreover, the polymerase can readily extend the dT opposite an *R*-cdA, but fails to extend the dT opposite an *S*-cdA, indicating that pol β stalls at an *S*-cdA following its incorporation of a dT. This further inhibits the ligation of the nick by DNA ligase I (LIG I), allowing flap endonuclease 1 (FEN1) to cleave nucleotides. Subsequently, this results in the gaps and accumulation of single-strand DNA break intermediates [84]. Thus, pol β bypass of an *R*-cdA can lead to nucleotide misincorporation causing mutations, whereas its bypass of an *S*-cdA can cause the accumulation of DNA strand break intermediates that in turn results in recombination and genome instability [84].

### 5.4. Repeat Sequence Instability Through the Bypass of cdA by Pol β

**Figure 9:**
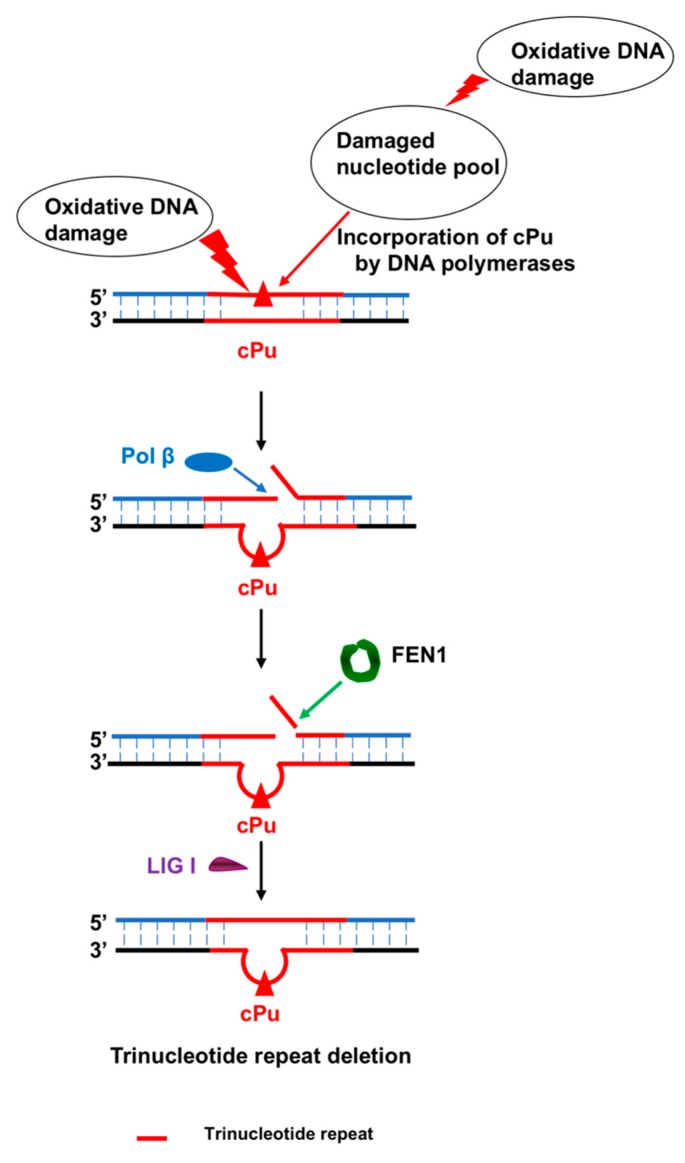
A cdA located at DNA repeat sequences induces repeat instability through pol β bypass of a loop structure. Pol β: DNA polymerase β; LIG I: DNA ligase I; FEN1: flap endonuclease 1.

Interestingly, although pol β stalls at an *S*-cdA that is located in the random sequences [84], it can efficiently extend a dT opposite to an *S*-cdA located in trinucleotide repeats, such as CAG repeats. This further results in CTG repeat deletion through BER [69]. It has been found that this is because both *R* and *S* diasteroisomers of cdA located on the template strand induce the formation of a CAG repeat loop in the template that mimics the intermediates formed during maturation of the lagging strand and BER [69]. This is likely due to the distortion of the backbone of the repeats, which subsequently induces the G:C self-base pair in the CAG repeats. Since pol β preferentially skips over a hairpin or loop structure [85,86], the loop structure in CAG repeats induced by a cdA lesion can also be readily bypassed by pol β, thereby leading to the displacement of the downstream repeat strand into a flap during DNA lagging strand maturation and BER [69]. Subsequently, the flap is captured and cleaved efficiently by FEN1, resulting in CTG repeat deletion (Figure 9). This is further supported by the fact that the locations of a gap relative to that of a cdA lesion in the CAG repeat template can govern the deletion of CTG repeats. A gap that is located upstream of or opposite to the lesion can result in CTG repeat deletion through the pol β bypass of a CAG repeat loop structure. However, a gap located downstream of the cdA that does not involve pol β loop bypass fails to cause repeat deletion [69]. These findings further demonstrate the essential role of pol β bypass of a loop structure containing a cPu lesion in mediating trinucleotide repeat deletion.

Although a study provides a mechanistic insight into the incorporation of cdATP by the Klenow fragment using superimposing modeling, crystallography-based structural studies on the Klenow fragment and other replication and repair polymerases and translesion DNA polymerases, especially eukaryotic DNA polymerases, are needed to understand the molecular mechanisms underlying cPu incorporation in DNA. Because DNA replication and repair polymerases often coordinate with their cofactors during DNA replication and repair, the effects of the coordination on the bypass of a cPu lesion and their impact on cellular function remain to be elucidated. Moreover, the effects of cPu lesions on the instability of repeated DNA sequences, including mono-, di-, tri-, tetra- and hexanucleotide repeats, through DNA replication and repair and the crosstalk among different DNA metabolic pathways and the underlying mechanisms need to be explored.

## 6. Quantification of cPu Lesions in DNA Samples

### 6.1. ^32^P-Postlabeling and Enzyme-Linked *Immunosorbent Assays*

A variety of analytical techniques for the quantification of oxidative DNA lesions in material derived from various types of cells and organisms have been developed, including radiolabeling approaches, enzyme-linked immunosorbent assays (ELISA), and chromatographic and mass spectrometry-based approaches, while the description of DNA damage (mitochondrial vs. nuclear) remains an important challenge [87]. It should be noticed that data obtained in different laboratories gave rise to different results, mainly due to deviations among experimental conditions used in various laboratories or inconsistent quantification of lesions because of oxidation artifacts [88,89]. Accurate quantification of cPu lesions is necessary to understand the biological relevance, and therefore analytical strategies must be set up in order to deliver reproducible and reliable results. Large discrepancies in the levels of the lesions determined by different groups are likely due to variations in analytical procedures, mainly in the steps of DNA extraction, hydrolysis of the nucleobases, and derivatization [30]. Each quantification technique is characterized by its own advantages, counterbalanced by some limitations for applicability to specific fields. The ^32^P-postlabeling approach for the measurement of cdA lesions developed by some research groups was important for increasing the detection limit to 1–5 lesions per 10^10^ unmodified nucleosides [90,91]. This methodology was then implemented for the identification and measurement of structurally diverse DNA carcinogen adducts [92], 8-oxo-dG in rat tissues [93], and oxidative DNA products, emerging as a suitable approach for detection of bulky lesions [94]. The levels of cdA in rat samples were significantly elevated in normal newborn samples compared to those in fetuses [90]. ^32^P-labeled adducts could be visualized by screen-enhanced autoradiography and quantified by Phosphorimager, Instant Imager, or Scintillation Analyzer [91,95].

Immunoassay methodologies are simple, fast, cost-effective, and reproducible for the identification of oxidatively induced DNA adducts within individual cells or tissues, while the combination of this approach with immunofluorescence, along with the presence of a monoclonal antibody specific for the lesion, enhanced the effectiveness of the method [96]. In the latter case, artifacts from interferences were eliminated, such as endogenous antibodies and cross-reactants, increasing the specificity and the sensitivity of this approach. The enzyme-linked immunosorbent assay (ELISA) has been shown to be successful in a wide variety of biological matrices, such as DNA, plasma, and urine, especially for the measurement of 8-oxo-dG, the most commonly investigated lesion as a biomarker of oxidatively induced DNA damage [97]. Recently, an immunoassay using a novel monoclonal antibody (CdA-1) specific for cdA in single-stranded DNA was generated [98]. The levels of cdA lesions measured through this ELISA indicated an accumulation of cdA with age in brain tissues of Xpa (-/-) mice compared with wild-type (wt) mice, and significantly, similar evidence was also revealed in liver and kidney tissues among these mice at 6, 24, and 29 months of age [99].

### 6.2. Liquid Chromatography Tandem Mass Spectrometry (LC–MS/MS)

Although ELISA and ^32^P-postlabeling methods represent applicable approaches with widespread use, owing to not requiring specialist experience and expensive equipment, lack of structural specificity seems to be the main problem of lesion overestimation compared with the concentrations measured by chromatography-based techniques coupled with isotope dilution mass spectrometric methodologies [100]. Indeed, the *m*/*z* values for parent and fragment ions contain important information about the structure of DNA lesions and the molecular composition, and when used in these approaches, are sufficiently specific for accurate quantification [101]. Gas chromatography/mass spectrometry (GC/MS) has been utilized to identify *R*-cdG and *S*-cdG in DNA, including a derivatization step by trimethylsilylation after the hydrolysis of the DNA sample [16,31,102]. However, this derivatization step may render the implementation of GC/MS methodology inapplicable due to artifacts at several stages of the analysis, likely due to the oxidation of the nucleobases present in acidic hydrolysis of DNA [103].

**Figure 10:**
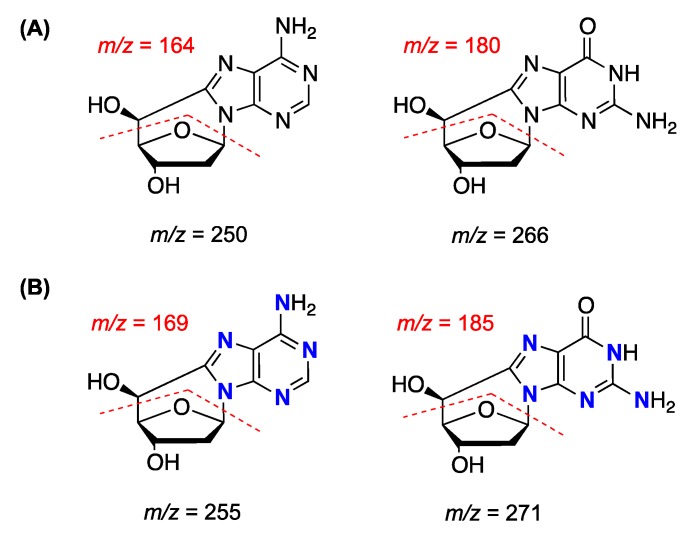
(**A**) MS/MS fragmentation spectra (ESI–MS/MS of the [M + H]^+^ ion) of *S*-cdA (*m*/*z* 250→164) and *S*-cdG (*m*/*z* 266→180) lesions. Similar fragment ions were observed for *R*-cdA and *R*-cdG lesions. (**B**) MS/MS fragmentation spectra (ESI–MS/MS of the [M + H]^+^ ion) of isotopically labeled (**N** = ^15^*N*) *S*-cdA (*m*/*z* 255→169) and *S*-cdG (*m*/*z* 271→185) lesions.

Liquid chromatography coupled with modern highly sensitive mass detectors, which follows a top-down approach, starting from the genetic material and going down to the single nucleoside level, is of great interest and extensively used due to its advantages in specificity and repeatability. On the contrary to immunoassays and the overestimation of specific DNA adducts [97], liquid chromatography–tandem mass spectrometry (LC–MS/MS) analysis ascertains accurate quantification of DNA adducts, providing lower limits the detection (LOD) and quantification (LOQ), thus improving the sensitivity of the methodology. Furthermore, the use of isotopically labeled reference compounds for the lesions enhances the reliability of the process, increasing the reproducibility and recovery of the quantification to a great extent. All these considerations make the LC–MS/MS approach an extremely important tool for the identification and estimation of oxidatively produced DNA products or adducts in relation to inflammatory and age-dependent disorders. The ion–current profiles of the corresponding mass transitions for *S*-cdA and *S*-cdG, which are acquired during LC–MS/MS analysis of the samples, are given in Figure 10. The ions of *m*/*z* 164 and 180 (together with 169 and 185 for the labeled cdA and cdG) result from the cleavage of both the *N*-glycosidic and the C4’–C5′ bonds of cPu. The stepwise procedure for the quantification of DNA lesions includes the isolation and purification of DNA from the biological specimen (biological fluid, cells, tissues, etc.) and the hydrolysis to single nucleosides by an enzymatic cocktail containing nucleases, followed by the analysis and quantification by liquid chromatography coupled with tandem mass spectrometry for the determination of the modified nucleosides. One of the problems for the exact quantification is the contamination of the genetic material before the enzymatic hydrolysis steps or by side products, such as those generated via oxidation of the unmodified nucleosides or/and degradation of the lesions (e.g., via oxidation of the lesions themselves), during the procedure. In order to avoid these sample workup artifacts, the presence of argon, metal chelators, and radical scavengers are added as part of the method [29,104].

**Figure 11:**
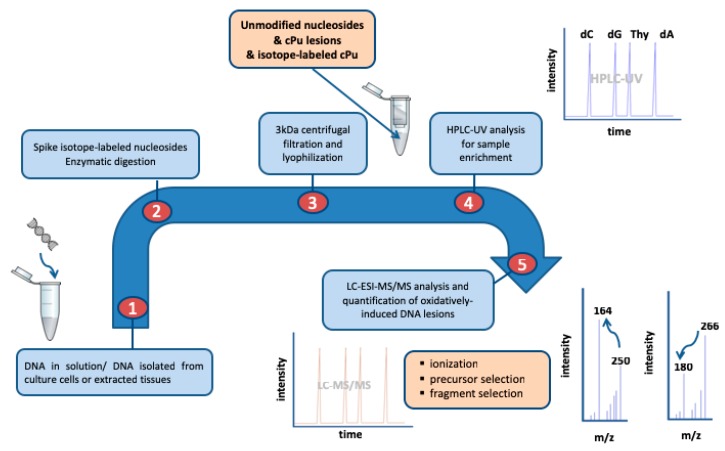
Workflow showing protocol steps for the quantification of cPu lesions via isotope dilution LC–ESI–MS/MS (the fragmentation pattern of the lesions is shown); dC: 2′-deoxycytidine; dG: 2′-deoxyguanosine; Thy: thymidine; dA: 2′-deoxyadenosine.

Two different approaches have been reported to date for the quantification of cPu via isotope dilution LC–MS/MS analysis. One approach is the direct injection of the sample into the LC–MS/MS system right after the enzymatic digestion step. The advantage of this methodology is the rapidity, as the unmodified nucleosides and the lesions are quantified together in one single run. However, in this approach, the samples do not pass through a purification step, and therefore materials that are incompatible with the mass detector materials, such as buffer ions carried from the enzymatic hydrolysis procedure, can create problems. Moreover, the unfiltered sample which is directly injected into the LC–MS/MS system comprises the unmodified nucleosides along with the lesions, resulting in a concentration which can be up to ∼60-fold in excess with respect to the lesions [105,106]. Another important consequence of the latter resulting in poor sensitivity is the limited solubility of the crude sample. A second approach for the quantification of DNA lesions is executed in two independent steps. Firstly, the sample is analyzed by an HPLC–UV system coupled with a sample collector, where the quantification of the unmodified nucleosides takes place based on their absorbance at 260 nm, whereas at the elution time-windows of the modified lesions, they are collected, pooled, and concentrated. In such an approach, the buffer ions carried from the enzymatic digestion step are separated from the analytes and the lesions from the unmodified nucleosides [29,30]. The sample containing the lesions can be further concentrated prior to LC–MS/MS analysis and each can be quantified by LC–MS/MS, increasing the overall sensitivity of the quantification method. An ameliorated enzymatic digestion protocol was developed based on the cost-effective genetically modified nuclease benzonase and the nuclease P1, which are necessary for the quantitative release of the cPu lesions [30]. Figure 11 illustrates a schematic representation of the isotope dilution LC–ESI–MS/MS chromatographic profile and the fragmentation pattern in the mass detector.

Comparison of cPu measured in an irradiated sample of calf thymus DNA by two different LC–MS/MS methods has been reported [107]. Data are obtained from the applied dose within the range of 0–60 Gy, and a linear dependence of lesions/10^6^ nucleosides vs. dose has been reported. The level of lesions/10^6^ nucleosides/Gy in γ-irradiated samples of calf thymus DNA in aqueous solutions, within the range of 0–60 Gy, was found to be 0.23 for cdA and 0.35 for cdG in ds-DNA using the method reported in Figure 11 [30], whereas the values of similar experiments were reported to be 14.2 for cdA and 20.1 cdG [105] using direct injection after the enzymatic digestion step without internal standards (i.e., spiked with known quantities of the isotopically enriched analytes), which is a ∼60-fold excess for both cdA and cdG.

## 7. Biological Studies for Health Applications

### 7.1. Neurological Diseases

Xeroderma pigmentosum (XP) is a rare disorder of defective UV radiation-induced damage repair that is characterized by photosensitivity and cancer of the UV-exposed areas of the skin and mucous membranes of the eyes and mouth at an early age. The skin is normal at birth, and with the onset of the disorder, all patients develop freckle-like pigmentary changes in sun-exposed areas, which eventually appear as poikiloderma [108,109]. XP can result from mutations in any of eight genes, denoted as XPA–XPG and XPV. Cells from the patients in complementation groups A through G are defective in nucleotide excision repair (NER) [110]. There is a relatively higher rate of XPA mutation in the Japanese population [111,112], which results in severe loss of neurons in multiple regions of the brain and spinal cord, with an age of onset between 7 and 13 years. The signs and symptoms of XP neurological disease include peripheral neuropathy, sensory neural deafness, microcephaly, cerebral dysfunction, ventricular dilation, cortical atrophy, and basal ganglia and cerebellar disturbances [113]. However, how the molecular defects in the NER pathway lead to the XP disease is far from being fully understood.

The first observations of the relationship between defective NER and XP neurological disease were provided by Robbins and coworkers, who examined the cell survival after exposure to UV radiation in patient-derived cells. They observed a correlation between the poor cell survival after UV exposure and the severity of neurodegeneration in the patients [114]. Robbins and colleagues hypothesized that since UV light cannot reach into the human brain, XP neurological disease results from specific endogenous DNA damages caused by free radicals, a kind of damage that is normally repaired by the NER pathway [115].

In the of NER deficiency, this induced damage can accumulate causing neuronal death by blocking transcription of essential genes. This study paved the way to consider other types of lesions caused by UV light, such as pyrimidine (6-4) pyrimidine photoproducts and cyclobutane pyrimidine dimers (CPDs) that block transcription, which are specifically repaired by NER and not by any other human enzymatic repair process. In this scenario, the cPu lesions can fulfill many of the criteria expected in neurodegenerative DNA lesions in XP. Specifically, these lesions are: chemically stable [116,117]; formed endogenously in mammalian cellular DNA [90,118]; repaired by the NER pathway and not by any other known process, as proven by several studies [39,44,55,119]; able to block strongly, but not completely, the transcription by RNA polymerase II that occurs in cells from XP patients [36,120].

These observations led some groups to propose that these lesions might be responsible, at least in part, for the neurodegeneration suffered by xeroderma pigmentosum (XP) patients, who lack the capacity to carry out NER [61,111,120]. Elucidating the mechanism by which cPu could have an implication in XP remains the main goal of several research groups. The studies carried out so far suggest three principal directions: (i) If the lesions completely block transcription by RNAPII, multiple genes are inactivated, and consequently, the levels of encoded proteins are reduced, causing neuronal death; (ii) If the lesions partially block transcription by RNAPII, mutant RNA transcripts can be produced, and this can result in mutant proteins that play a role in neuronal death [121]. In addition, the presence of cPu-induced distortion in the DNA structure prevents the binding of transcription factors (TF) [75,122] to DNA, causing reduced or dysregulated gene expression, in turn resulting in neuronal death; (iii) Other possible implications of the presence of cPu lesions in XP have been hypothesized by Arczewska et al. [123], who suggest that cPu in XP may impair the ability of BER proteins or other repair proteins to bind to cPu lesions, block transcription, and trigger transcriptomic reprogramming.

Recent studies have demonstrated the ability of poly(ADP-ribose) polymerase 1 (PARP-1), responsible for maintaining the integrity of the genome, to recognize and bind DNA sequences containing *S*-cdA and *R*-cdA [40]. DNA recognition and repair proteins other than NER may bind to such lesions but fail to repair them. This may be relevant in understanding the potential role of these lesions in a number of neurological diseases.

A growing body of evidence [124] indicates that NER-defective diseases, besides presenting the accumulation of nuclear DNA lesions, present high ROS levels, mitochondrial dysfunction, and increased reliance on glycolysis. Recent evidence [125] indicates that mitochondrial dysfunction is a neglected but important component of the DNA repair-defective syndromes. Fang et al. found that the neurodegeneration of XPA patients may be associated with mitochondrial and mitophagic dysfunction through PARP-1 hyperactivation and NAD*/SIRT1 reduction [126]. Moreover, this study suggested that the dysfunction was in common with other neurodegenerative disorders, such as ataxia–telangiectasia (AT) and Cockayne syndrome (CS). However, it is possible to consider that because of the differences in neurodegeneration in XPA, AT, and CS, that different mechanisms are involved in these diseases. CS is a rare autosomal recessive disease caused by mutation of either of two genes (CSA or CSB) critical for a subpathway of NER, termed transcription-coupled NER (TC-NER), which preferentially deals with transcription-blocking (typically bulky) lesions within active regions of the genome. The neurological abnormalities that affect CS patients are qualitatively different from those seen in NER-deficient XP patients. In XP, the neurons are primarily affected. In contrast, in CS, the disease involves primarily the white matter of the brain and features brain calcification as well as vascular abnormalities, neither of which are ever observed in XP neurologic disease [61,127]. It was found that CSA is involved in the repair of *S*-cdA and 8-oxo-dG [128].

The differences between CS and XP neurological diseases reflect fundamentally different underlying mechanisms [61,129]. Mitochondrial dysfunction might be a primary or a secondary causative effect in these diseases, depending on the specific DNA repair defect; further evidences will be needed to prove that the accumulation of endogenous cPu lesions in the brain plays a causative role in XP neurological disease [99] and is involved mitochondrial and mitophagic dysfunction.

### 7.2. Cancer and Aging

**Figure 12:**
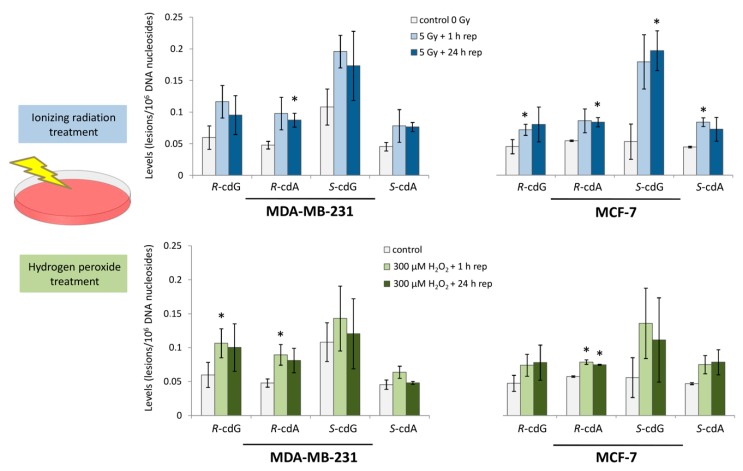
Levels of cPu lesions/10^6^ nucleosides measured by LC–MS/MS from breast cancer MDA-MB-231 and MCF-7 cells with or without exposure to γ-irradiation (upper part) and hydrogen peroxide (lower part). The light bars (blue and green, respectively) represent samples exposed to 5 Gy or 300 μΜ H_2_O_2_ followed by a 1 h repair period, and the dark bars (blue and green, respectively) represent samples exposed to 5 Gy or 300 μΜ H_2_O_2_ followed by a 24 h repair period. The white bars represent untreated samples (control). The asterisks denote a statistically significant difference (*p* < 0.05) between the untreated controls and the treated samples. From reference [133].

Carcinogenesis is a complex process characterized by a progression of abnormalities over time before a cell becomes malignant, while a variety of DNA mutations play crucial roles in the process development. Increased rates of formation of ROS and their interaction with genetic material lead to high yields of oxidatively induced nucleoside modifications [130]. Inability to efficiently repair these harmful species in the genome may affect DNA integrity, causing genetic instability and enhancing cancer risk [131,132]. The levels of cdA and cdG, in their *R* and *S* diastereoisomeric forms, have been identified in estrogen receptor-alpha (ER-α) MCF-7 and triple-negative MDA-MB-231 breast cancer cells upon exposure to two distinct conditions of radical stress, i.e., 5 Gy of ionizing radiation and 300 μM hydrogen peroxide, followed by an interval period that allows DNA repair [133]. The isotope dilution LC–ESI–MS/MS analysis revealed that ER-α MCF-7 and MDA-MB-231 cells are highly susceptible to radiation-induced DNA damage (Figure 12). Estrogen-induced ROS formation can stimulate oxidative DNA damage, while differences in NER efficiency of the breast tissue strongly support the association between modified DNA nucleobases and breast cancer repair capacity [134,135]. This was the first study reporting the simultaneous quantification of the four cPu lesions of DNA in human breast cancer cell lines before and after treatment with DNA-damaging agents. Upon exposure to a dose of 5 Gy, it was found that *S*-cdG was the most abundant lesion, with detected levels in the range of 0.03–0.14 to 0.17–0.23/10^6^ nucleosides in both cell lines, whereas *R*-cdG in MCF-7 and *R*-cdA in MCF-7 and MDA-MB-231 cells were indicated as being significantly increased with respect to untreated cell cultures. Exposure of cells to hydrogen peroxide revealed that the *S*-cdG was still observed as the most abundant, and statistically significant differences were obtained at the levels of *R*-cdA in both cell lines along with the levels of *R*-cdG in MDA-MB-231 cells [133].

Previous studies indicated the elevated levels of *S*-cdA in malignant HCC1937 and MCF-7 breast cancer cells after exposure to hydrogen peroxide (0.04–0.06/10^6^ nucleosides) [136]. Moreover, the levels of *R*-cdG, *S*-cdG, and *S*-cdA were found to be increased in cultured lymphoblasts of women with BRCA1 mutations after exposure to 5 Gy of ionizing radiation followed by a subsequent 1 h period of cellular repair of this damage (0.2–0.3/10^6^ nucleosides, 0.6–0.8/10^6^ nucleosides, and 0.2–0.7/10^6^ nucleosides, respectively) [137]. Increased levels of cdA and cdG have been observed in organs of prdx1^+/+^ and prdx1^−/−^ mice in a model study of the tumor susceptibility in prdx1^−/−^ animals and the link with oncogenes, such as c-Myc and DNA damage [138]. Furthermore, elevated cPu levels have been measured in the skin of red Mc1r^e/e^ mice compared with albino Mc1r^e/e^ mice in a study of the mechanism of UV-independent carcinogenesis aiming to ascertain whether ROS-mediated oxidative DNA damage is influenced by the pheomelanin synthesis pathway [139].

**Figure 13:**
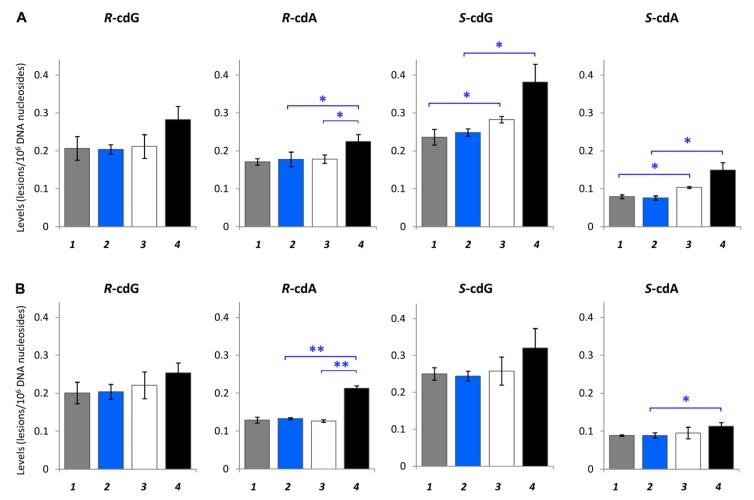
Levels of cPu lesions/10^6^ nucleosides measured by LC–MS/MS in control severe combined immunodeficient (SCID) and tumor-bearing SCID mice. (**A**) Levels of *R*-cdG, *S*-cdG, *R*-cdA, and *S*-cdA lesions in genomic DNA isolated from the liver of control SCID mice (group ***1***: 4 weeks old; group ***2***: 17 weeks old) and tumor-bearing SCID mice (group ***3***: 4 weeks old; group ***4***: 17 weeks old). (**B**) Levels of *R*-cdG, *S*-cdG, *R*-cdA, and *S*-cdA lesions in genomic DNA isolated from the kidney of control SCID mice (groups ***1*** and ***2***) and tumor-bearing SCID mice (groups ***3*** and ***4***). * Denotes a statistically significant difference (*p* < 0.05) and ** denotes a statistically significant difference *p* < 0.005 between the animal groups. From reference [140].

Recently, the accumulation of both the *R* and *S* diastereoisomers of cPu was followed up in organs of tumor-bearing severe combined immunodeficient (SCID) mice of different ages (4 and 17 weeks old) in comparison with the corresponding control SCID mice, providing evidence of increased oxidatively induced DNA damage occurring during tumor progression [140]. Identification and quantification by isotope dilution LC–ESI–MS/MS in two distinct tissues (the liver and kidney) revealed that tumor-bearing SCID mice had 1.1–1.4-fold higher levels of the four cyclopurines than the control SCID mice, while *S*-cdG was the most abundant lesion, with levels of 0.28–0.38/10^6^ nucleosides in the liver and 0.24–0.25/10^6^ nucleosides in the kidney, respectively (Figure 13). Moreover, in 17-week-old tumor-bearing animals, the cPu lesion levels were elevated compared to 4-week-old tumor-bearing mice, in agreement with previously reported data among cancer and age-dependent processes [141]. The total amount of cPu lesions in both tissues for all groups are reported in Table 2. Young and old control SCID mice presented similar levels of cPu (0.70/10^6^ nucleosides in the liver and 0.67/10^6^ nucleosides in the kidney). Among young mice, the diseased ones showed 1.1-fold higher cPu levels. Between the early and latest stages of the lifespan of tumor-bearing SCID mice, increased damage is observed at the levels of 1.04/10^6^ nucleosides in the liver and 0.9/10^6^ nucleobases in the kidney of 17-week-old diseased mice. Important evidence also can be provided by the ratios of *R*/*S* for both cdG and cdA in different tissue compartments. The *R*/*S* ratios were found to be almost the same in the liver and kidney for all five experimental animal models for cdG (0.7–0.9). On the contrary, the ratio for cdA was approximately 1.3–1.9 in the kidney and 1.5–2.3 in the liver, being twice than that of cdG [140].

In Table 2, the diastereoisomer ratios for the different organs of control SCID and tumor-bearing SCID mice are summarized. The diastereomeric ratio (*R*/*S*) attracts interest since it can inform on mechanistic issues. It is worth underlining that the *S* form is always more abundant than the *R* form in cdG, whereas in cdA, the *R* form is always more abundant than the *S* form. These cyclopurine isomer ratios can be explained by the influence of at least two factors: (i) the local conformations of the supramolecular organization of DNA are taken at the reactive sites prior to C5′ radical cyclization, which make one type of diastereoisomer more prevalent, and (ii) the NER efficiency for the repair of the diastereoisomers. Indeed, previous work in human HeLa cell extracts indicated that the cdA and cdG lesions are excised with similar efficiency by NER and that the *R*-diastereoisomers of both cdA and cdG cause greater distortion of the DNA backbone and are better substrates of NER than the corresponding *S* ones [39].

Increased levels of cPu lesions were observed in animal tissues and biological fluids at background levels or at increased levels in age-related disorders as well as in other inflammation-related pathologies. Accumulation of cPu lesions with aging in a tissue-specific manner (liver > kidney > brain) of wild-type and DNA repair-deficient progeroid ERCC1^−/Δ^ mice is also observed, indicating the significant higher levels of cdA and cdG in ERCC1^−/Δ^ mice compared to age-matched wt mice [118,142]. NanoLC–ESI–MS/MS analysis of liver tissues of Long–Evans Cinnamon (LEC) rats, as an animal model of human Wilson’s disease, and Long–Evans Agouti (LEA) healthy rats demonstrated significant elevated levels of cdA and cdG in both *R* and *S* isoforms in diseased animals (up to 4-fold excess for liver tissues of LEC^+/−^ and brain tissues of LEC^−/−^) [29,143]. In Table 2, the *R*/*S* levels of cdG and cdA lesions in tissues of selected animal models associated with age-related processes are also reported. It is worth recalling that the *R*/*S* ratios can be used to support further biological implications in the formation and/or repair of these lesions, as discussed above. However, comparing all the data reported in Table 2, in some studies, a clear scenario of the *R*/*S* formation cannot be drawn, probably due to the analytical performance and uncertainty of the measurements. It would be recommended to reach uniformity of protocols and quantitative methods in order to diminish these pitfalls. It is worth mentioning that previous studies using different analytical tools, such as GC/MS or LC/MS, indicated the accumulation of *R*-cdA and *S*-cdA in liver DNA of neil1^−/−^ mice not exposed to exogenous oxidative stress, revealing that NEIL1 plays a role in the cellular repair of *R*-cdA and *S*-cdA [144]. Previous studies by the same group on this direction highlighted elevated levels of *S*-cdA in organs of csb^−/−^ mice compared to wild-type mice, providing the evidence that CSB is implicated in the repair of the DNA helix-distorting tandem lesion *S*-cdA [145] as well in tissues of prdx1^−/−^ mice with approximately identical *R*-cdG/*S*-cdG and *R*-cdA/*S*-cdA ratios (for cdG, it was found to be 0.23, 0.40, and 0.17, while for cdA, 0.11, 0.08, and 0.23 in the brain, liver, and spleen, respectively) [138]. Lastly, increased levels of *S*-cdA were found in polymorphonuclear leukocytes of familial Mediterranean fever (FMF) patients when compared to control subjects [146], while *R*-cdA and *S*-cdA were found to be elevated in urine samples of prediabetic patients [147].

### 7.3. Addendum

During the revision process of this review, a “Letter to the editor” was published in *Free Radical Research* by Cadet et al. [148] criticizing the work produced by several laboratories [29,89,133,138,142,148] and described here. In particular, Cadet et al. [148] questioned the effectiveness of the published protocol on DNA samples shown in Figure 11 and of the LC–MS/MS methodologies used for detection and quantification of cPu as biomarkers of DNA oxidation reactions. In their Letter, the authors did not present any new experimental work, but only gave a “revisited analysis” of DNA damage detection based on some unsuccessful attempts (Cadet et al., unpublished) made in 2000 to measure the presence of cPu lesions in DNA by LC–ESI–MS/MS, using either the brain of NER-knockout mice or gamma-irradiated human cells exposed to 1 kGy radiation. Therefore, based on a unique unsuccessful study of almost twenty years ago, in their review, the authors started to cast doubts on peer-reviewed work published in the last ten years from several important laboratories around the world and advanced the hypothesis that cPu formation can be an experimental artifact. Apart from the fact that the isotope dilution LC–MS/MS methodology is a sensitive methodology that nowadays overcomes problems of low-sensitivity methods faced twenty years ago, the artifact formation in the analysis of biological samples cannot be underestimated, especially after the well-known debate of some years ago on similar problems raised with 8-oxo-dG lesion detection. Indeed, this review describes the experience acquired in the last years for new and effective protocols of digestion and LC–MS/MS analysis of DNA samples, highlighting the importance of quantitation sensitivity, accuracy, precision, linearity, reproducibility, and recovery, along with the DNA enzymatic digestion efficiency. The synthesis of ^15^N isotopically labeled compounds for each of the four cPu diastereoisomers was also an important step that allowed to establish an analytical protocol with an HPLC cleanup and enrichment of the samples (Figure 11). Therefore, to answer critical questions referring to supposed experimental artifacts, the effort and time that must be spent is such that only a few labs can afford. By using control experiments and DNA analyses of samples before and after irradiation, the criticisms on artifacts could be successfully overcome, and the resulting robust analytical methodology can be nowadays used to gain information on the importance of cPu as biomarkers of hydroxyl radical damage in aging and diseases. This addendum is made to trace the evolution of cPu research over time and also to offer some arguments for debating how the analytical protocols can be developed and ameliorated over time.

## 8. Conclusions

In this review, we have summarized the recent work on purine 5′,8-cyclo-2′-deoxynucleosides (cPu), a subject of growing interest in studies on DNA damage and impact on human health and disease. The cPu lesions are forms of oxidative DNA damage that can be repaired by NER with low efficiencies. The accumulation of cPu lesions in the genome inhibits the DNA replication by diminishing the activities of repair polymerases, RNA polymerases, and the DNA binding of transcription factors. This results in severe adverse effects on cellular functions, including replication fork stalling, deficient DNA repair and gene transcription, mutagenesis, and genomic instability.

The use of these lesions as candidate biomarkers of DNA damage is increasingly appreciated because the cPu DNA lesions do not suffer from stability issues and artifacts of other oxidatively generated DNA lesions. Moreover, an interesting feature of the cPu lesions is that they exist in four diastereoisomeric forms in different ratios. The presence of these DNA lesions in different tissue-types and biological environments is well-documented. However, the impact of the stereoisomeric cPu lesions on various pathologies is poorly understood, and thus new knowledge needs to be developed in order to achieve deeper insights into these phenomena. The state of the art described in this review indicates that important steps have been made for clarifying many of the of biochemical and biological processes involving cPu lesions. These achievements will certainly facilitate further extended analytical studies of the detection of these modified nucleosides in vivo and the assessment of their impact on genome integrity, human health, and aging.

## Figures and Tables

**Table 1:** Melting points (Tm) of a series of double-stranded (ds) oligonucleotide sequences.

Length	Sequence	Tm, °C	Reference
*11-mer*	5′-d(CGT AC**X** CAT GC)-3′		[37]
	3′-d(GCA TG**Y** GTA CG)-5′		
	**X** = dA, **Y** = T	49.5	
	**X** = *S*-cdA, **Y** = T	42.0	
*12-mer*	5′-d(GTG C**X**T GTT TGT)-3′		[38]
	3′-d(CAC G**Y**A CAA ACA)-5′		
	**X** = dG, **Y** = C	55.0	
	**X** = *S*-cdG, **Y** = C	46 ± 1	
*14-mer*	5′-d(ATC GTG **X**CT GAT CT)-3′		[24]
	3′-d(TAG CAC **Y**GA CTA GA)-5′		
	**X** = dA, **Y** = T	54 ± 1	
	**X** = *S*-cdA, **Y** = T	48 ± 1	
*17-mer*	5′-d(CCA CCA AC**X** CTA CCA CC)-3′		[39]
	3′-d(GGT GGT TG**Y** GAT GGT GG)-5′		
	**X** = dA, **Y** = T	65.2 ± 0.6	
	**X** = *R*-cdA, **Y** = T	58.9 ± 0.6	
	**X** = *S*-cdA, **Y** = T	60.5 ± 0.6	
	**X** = dG, **Y** = C	66.2 ± 0.7	
	**X** = *R*-cdG, **Y** = C	63.4 ± 1.0	
	**X** = *S*-cdG, **Y** = C	63.5 ± 0.6	
*23-mer*	5′-d(GCA GAC ATA TCC TAG AG**X** CAT AT)-3′		[40]
	3′-d(CGT CTG TAT AGG ATC TC**Y** GTA TA)-3′		
	**X** = dA, **Y** = T	60.0 ± 0.3	
	**X** = *R*-cdA, **Y** = T	59.0 ± 0.2	
	**X** = *S*-cdA, **Y** = T	58.0 ± 0.3	

**Table 2:** Diastereomeric ratios (*R*/*S*) of cdG and cdA lesions in animal tissues by isotope dilution liquid chromatography–tandem mass spectrometry ^#^.

DNA Source	*R*-cdG/*S*-cdG	*R*-cdA/*S*-cdA	Reference
*Liver*			[140]
normal Swiss mice 4w	0.85	1.81	
normal Swiss mice 17w	0.79	1.77	
control SCID mice 4w	0.87	2.16	
control SCID mice 17w	0.82	2.34	
tumor-bearing SCID mice 4w	0.75	1.73	
tumor-bearing SCID mice 17w	0.74	1.51	
*Kidney*			
normal Swiss mice 4w	0.86	2.03	
normal Swiss mice 17w	0.77	1.72	
control SCID mice 4w	0.80	1.45	
control SCID mice 17w	0.84	1.49	
tumor-bearing SCID mice 4w	0.85	1.56	
tumor-bearing SCID mice 17w	0.79	1.88	
*Liver*			[118,142]
wild-type mice 10w	0.65	0.95	
wild-type mice 21w	0.96	1.64	
ERCC^−/Δ^ mice 10w	1.20	2.65	
ERCC^−/Δ^ mice 21w	1.30	2.05	
*Kidney*			
wild-type mice 10w	0.39	0.77	
wild-type mice 21w	0.22	0.38	
ERCC^−/Δ^ mice 10w	0.53	1.42	
ERCC^−/Δ^ mice 21w	0.26	0.40	
*Brain*			
wild-type mice 10w	0.45	0.64	
wild-type mice 21w	0.29	0.67	
ERCC^−/Δ^ mice 10w	0.32	0.81	
ERCC^−/Δ^ mice 21w	0.44	0.90	
*Liver*			[29,143]
LEA rats 3m	0.68	0.91	
LEC^+/−^ rats 3m	0.77	1.29	
LEC^+/−^ rats 12m	0.50	0.86	
LEC ^−/−^ rats 1m	0.80	1.75	
LEC^−/−^ rats 3m	0.79	0.96	
LEC^−/−^ rats 6m	0.65	1.6	
*Brain*			
LEA rats 3m	0.88	1.70	
LEC^+/−^ rats 3m	0.81	2.17	
LEC^+/−^ rats 12m	1.61	2.80	
LEC^−/−^ rats 1m	1.00	1.60	
LEC^−/−^ rats 3m	0.73	1.33	
LEC^−/−^ rats 6m	1.21	2.15	
*Skin*			[139]
red-*Mc1r^e/e^*mice	0.40	0.86	
albino-*Mc1r^e/e^*mice	0.42	1.20	

#w: weeks; SCID: severe combined immunodeficient; ERCC: Excision repair cross-complementing; LEA: Long–Evans Agouti; LEC: Long–Evans Cinnamon.

## Abbreviations

BER	base excision repair
CS	Cockayne syndrome
cdA	5′,8-cyclo-2′-deoxyadenosine
cdG	5′,8-cyclo-2′-deoxyguanosine
cPu	purine 5′,8-cyclo-2′-deoxynucleoside
dA	2′-deoxyadenosine
dG	2′-deoxyguanosine
LC–MS/MS	liquid chromatography tandem mass spectrometry
LEA	Long–Evans Cinnamon
NER	nucleotide excision repair
ODNs	oligodeoxynucleotides
ROS	reactive oxygen species
SCID	severe combined immunodeficient
Pol	DNA polymerase
Dpo4	DNA polymerase IV of *Sulfolobus solfataricus*
LIG I	DNA ligase I
TBDMS	tert-butyldimethylsilyl
XP	xeroderma pigmentosum
